# A novel technique for endoscope progression in gastroscopy resection: forward-return way for dissection of stromal tumor in the muscularis propria of the gastric fundus

**DOI:** 10.3389/fonc.2023.1077201

**Published:** 2023-05-18

**Authors:** Hai-Mei Guo, Ying Sun, Shuang Cai, Feng Miao, Yan Zheng, Yang Yu, Zhi-Feng Zhao, Lu Liu

**Affiliations:** Department of Gastroenterology, The Fourth Affiliated Hospital of China Medical University, Shenyang, Liaoning, China

**Keywords:** gastrointestinal stromal tumor, Forward-Return Way, muscularis propria, gastric fundus, common submucosal tumor

## Abstract

**Background:**

The fundus of the stomach is a challenging region for endoscopic resection of gastrointestinal stromal tumors (GISTs), especially in the anterior wall of the fornix at the side of the greater curvature. This study aimed to introduce the Forward-Return Way (FRW) technique in gastric fundus operations and provide evidence of its advantages. The FRW technique allows the gastroscope to access the stomach fornix without entering the gastric antrum after passing through the gastric cardia. Using FRW, the gastroscope body makes a forward return along the wall of the posterior wall of the upper gastric body and the wall of the greater curvature.

**Methods:**

The clinical data of patients with stromal tumors in muscularis propria at the gastric fundus (STMF) at the Fourth Hospital of China Medical University between May 2020- March 2021 were reviewed. The novel FRW technique was used in the procedures, and the beneficial effects, suitability, applicable lesion site, and success rates of FRW were analyzed.

**Results:**

A total of 10 cases were reviewed, and the FRW technique was successfully performed in 7 cases (70%). The gastroscope’s tip reached the area just below the gastric cardia, allowing endoscopists to successfully access all angles and sites of the stomach’s fundus in all seven patients. The lesion was easily accessed, and the gastroscope was stable with good left-right and forward-backwards movements.

**Conclusion:**

The FRW technique significantly facilitates the resection of the GISTs by aligning the endoscopy body movement direction with the observation direction. Gastrointestinal Stromal Tumor; forward-return of gastroscopy along the gastric body wall; muscularis propria; gastric fundus.

## Background

Gastrointestinal stromal tumors (GISTs) are the most common gastrointestinal mesenchymal neoplasms and one of the clinically common submucosal tumors (SMT) of gastric origin (1, 2). Stromal tumors in muscularis propria (STMF) occur more at the fundus of the stomach, accounting for 51.5% of all GISTs sites (3, 4). The clinical presentation of STMF is nonspecific, with only a few patients experiencing gastrointestinal bleeding (5). Currently, complete resection of STMF is the best treatment (6), among other approaches, including traditional open surgery, laparoscopy, and gastroscopy dissection. However, many reports have demonstrated that gastroscopy dissection is the safest and most effective treatment for STMF of the fundus (4).

Several gastroscopy dissection techniques are used nowadays in clinical practice, including Endoscopic submucosal dissection (ESD) (7), endoscopic submucosal excavation (ESE) (8), endoscopic full-thickness resection (EFTR) (9, 10), submucosal tunnelling endoscopic resection (STER) (11), and the combination of EFTR and laparoscopic approach (4, 12–15). The fundus of the stomach is a challenging area for endoscopic resection of tumors in muscularis propria (16), especially when using ESD (17), ESE, or EFTR (18, 19). Additionally, endoscopic resection of lesions in the middle of the fornix and the anterior wall of the fornix is extremely difficult in clinical practice, especially the anterior wall of the fornix at the side of the greater curvature.

In some STMF cases, the removal procedure requires a U-turn of the distal tip of the gastroscope (20). In these cases, the progress of the tip is opposite to the moving direction of observation, and the body of the gastroscopy becomes suspended without support, making it difficult to operate and control. In 2016, Professor Zhi-Feng Zhao discovered the Forward-Return Way technique (FRW) during the endoscopic treatment of a patient with STMF and successfully completed the procedure. The FRW technique allows the gastroscope to access the stomach fornix without entering the gastric antrum after passing through the gastric cardia. Using FRW, the gastroscope body makes a forward return along the wall of the posterior wall of the upper gastric body and the wall of the greater curvature. The present study introduces this new endoscopy progression technique and the results of its applicability in practice.

## Patients and methods

### Subjects

The clinical data of STMF patients who accepted the new FRW technique at the Fourth Hospital of China Medical University from May 2020 to March 2021 were retrospectively analyzed. This study has been approved by our hospital’s Ethics Committee (Ethics Approval No.: EC-2021-HS-001). All patients have been informed about the FRW technique and given their written consent before the procedure. This study was a retrospective study, not a clinical trial, so we did not register it on clinicaltrials.gov or other similar websites. All authors had access to information that could identify all the patients during or after data collection.

Case selection requirements: (i) gastroscopy revealing elevated mucosa at the fundus of the stomach; (ii) endoscopic ultrasonography revealing STMF; (iii) no history of gastric cardiac or gastric surgery; (iv) tolerance to general anaesthesia and tracheal intubation; (v) lesions diameter of ≤5 cm (21); (vi) patients who were informed with the new endoscopic technique and signed the consent form.

Exclusion Criteria (contraindications for endoscopic resection of gastrointestinal submucosal tumors (21)) were: (i) when an enlarged lymph node or distant metastasis lesion was identified; (ii) patients with a poor general health condition who could not tolerate endoscopic surgery; (iii) patients with bleeding and/or ulceration on the surface of the lesion; (iv) patients whose pathology results showed they were non-GIST patients.

## Methods

### The endoscopy progression technique

**Figure 1:**
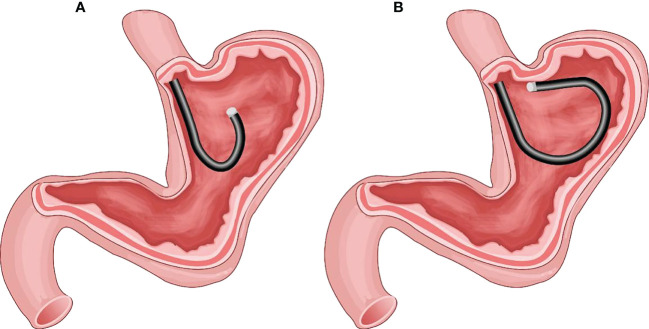
**(A)** The gastric fundus and cardia as observed by the traditional U-turn technique; the gastroscopy body is suspended without support. **(B)** Endoscopy progression just below the gastric cardia using the forward-return of gastroscopy along the greater curvature wall using the FRW technique; the gastroscopy body is supported by the gastric wall.

The FRW technique enables the gastroscope to pass through the gastric cardia without entering the gastric antrum while the endoscopy progresses continuously. The gastroscope body makes a forward return along the wall of the posterior wall of the upper gastric body and the wall of greater curvature to access the fornix of the stomach. When the gastroscope pushes forward, its tip moves towards the fundus of the stomach, and when the gastroscope pulls back, its tip moves away from the fundus. Thus, the gastroscope body is fixed in the upper gastric body by the stomach wall without being suspended. We called this endoscopy progression technique: Forward-Return Way (FRW). This new technique avoided the paradoxical movement in other advanced endoscopy progression skills, mainly the common U-turn. (Please refer to **Figure 1**).

### Endoscopic resection

All endoscopic surgeries were performed following Professor Zhi-Feng Zhao’s guidance on the FRW technique. The operators were senior endoscopists in our endoscopic treatment center with several years of experience in EFTR surgery. The patients underwent endotracheal intubation under intravenous anaesthesia before the endoscopic surgery. The anaesthesia of choice for the endoscopic surgeries of STMF was CO2 (22, 23). For the endoscopic resection of STMF, we mainly used EFTR (24).

### Data collection

**Figure 2:**
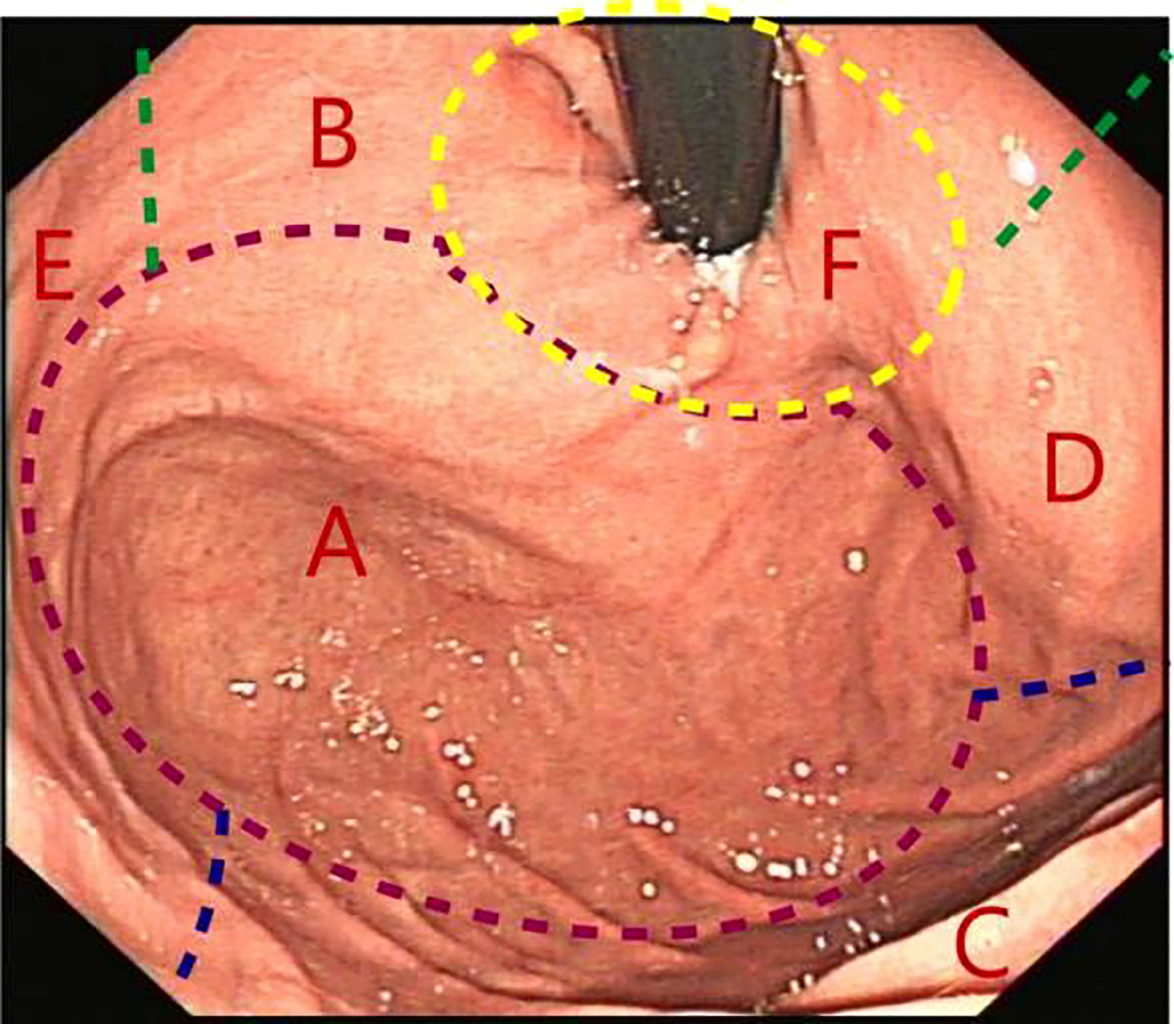
Gastric fundus subdivisions. **(A)** Fornix; **(B)** Lesser curvature of the stomach; **(C)** Greater curvature of the stomach; **(D)** Anterior wall; **(E)** Posterior wall; **(F)** Gastric cardia.

The following data were collected and analyzed: (i) basic information of patients; (ii) whether the FRW technique was successfully performed; and (iii) the selected endoscopic method (after successful FRW). The lesion sites suitable for the FRW technique were selected according to the division of the gastric fundus and the lesion area, as shown in **Figure 2**. Moreover, the success rate, results, and operation characteristics of the FRW technique were analyzed.

Manipulating performance evaluation criteria after FRW implementation (**Table 1**)

**Table 1:** Manipulating performance evaluation criteria after FRW implementation.

Score	The direction of endoscopy body movement is the same as the direction of observation	Ease of instrument entry and exit	The flexibility of endoscopy body control	The ability to access and observe the lesion	Stabilization degree of endoscopy body at fixation and movement
2	Fully achieved	Easy (all devices pass easily and entry and exit freely)	Excellent (endoscopy body moves freely; various endoscopic movements are the same as in other parts)	Excellent (clear and stable visualization, like conventional visualization at other sites; various observations can be achieved)	Excellent (endoscopy body does not shake, almost unaffected by respiration or heartbeat.)
1	Partial achieved	Acceptable (various devices can pass through, but some devices have significant resistance when passing through)	Acceptable (there is instability in some directions of movement, but it meets the manipulating requirements)	Acceptable (Unstable visualization, but can perform various endoscopic procedures)	Acceptable (there is uncontrolled movement of the endoscopy body during the procedure, or it is influenced by respiration and heartbeat, but the procedure can be completed)
0	Not achieved	Not feasible (some instruments cannot pass through)	Poor (Severe limitation of endoscopy movement causing many procedures to be impossible)	Poor (difficult to maintain stable visualization, severely affecting manipulating, or unable to observe details during the procedure)	Poor (endoscopy body shaking, affected by breathing and heartbeat, or difficult to manipulate)

### Postoperative management

After the EFTR procedure, patients were confined to bed rest, food fasting, and water fasting for three days. The above measures and gastrointestinal decompression were performed for five days after EFTR. Parenteral nutrition was given during fasting, and continuous oral proton pump inhibitor and gastric mucosal protective agents were administered 6-8 weeks postoperatively. Patients were observed for complications of EFTR, including bleeding, perforation, and infection (25).

### Follow-up

All patients were followed up regularly with gastroscopy examinations at 3, 6, and 12 months after the operation, and then they were checked once a year to monitor wound healing and to look for any residual or recurrent tumors.

### Histopathological evaluation

H&E staining and immunohistochemical staining were conducted to identify the nature of the lesion and to examine any tendency to malignant transformation.

### Statistical analyses

Statistical analyses were performed using IBM SPSS Statistics 25.0 (SPSS Inc., Chicago, IL, USA). Continuous data were expressed as `*X* ± *s*. Categorical data were presented as numbers or percentages (%). *FRW handling performance score was expressed as X ± s.*

## Results

### Patient information

A total of 112 cases diagnosed with STMF and treated in the Fourth Affiliated Hospital of China Medical University from 2016 to 2020 were evaluated. Ten patients qualified for the FRW technique (2 males and 8 females), aged 57.6 ± 10.07 (46-78), with a lesion diameter of 0.5-5.0cm. The FRW technique was successfully performed in 7 patients (70%) and failed in 3 patients (30%). (See **Table 2**).

**Table 2:** Case information.

Items	Details	Number of cases
Age	57.6 ± 10.07 (46-78) years	10
Sex	Male	2
	Female	8
Lesion diameter	0.5-5.0 cm	
Location	Middle of the fornix	1
	Greater curvature side of the anterior wall of the fornix	4
	The posterior wall of the fundus	1
	Greater curvature side of fundus	1
	Greater curvature side of the middle of the fornix	3
Successful FRW	7/10	70%
Surgical duration:	47-122 min	
Complications:	None observed	0
Length of hospitalization	7.29 ± 0.49 (7-8) days	

### The results of the FRW technique application

Using the FRW technique, the tip of the gastroscope could access the location just below the gastric cardia in 7 out of 10 patients. All angles of the fundus of the stomach were operable in all 7 patients. Moreover, the FRW technique made accessing and observing the lesion much simpler without the need for paradoxical movement.

### The suitable location for the FRW technique

**Figure 3:**
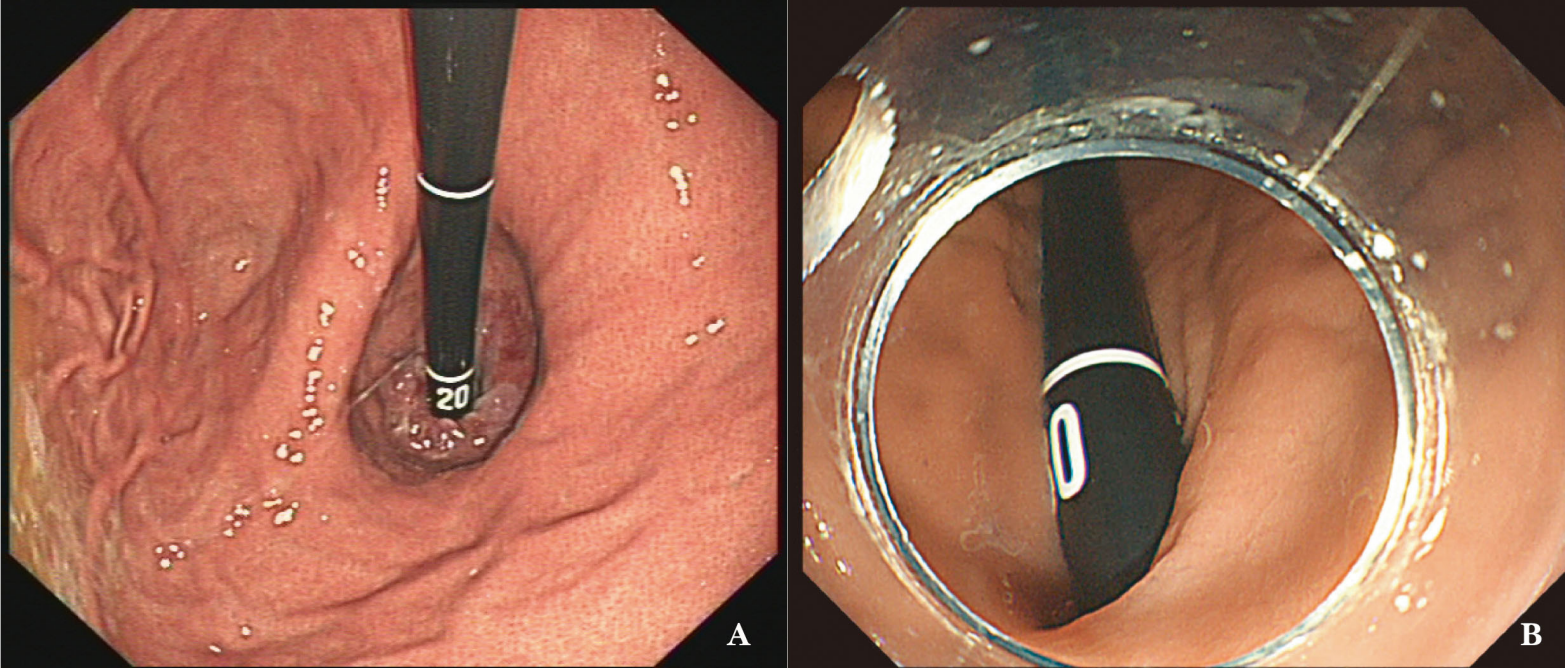
Two views of accessing subcardia using two methods. **(A)** Conventional metho**(D)** Due to the U-shaped reversal of the gastroscope and the limitation of the maximum angle of the gastroscope body, the view is directly facing the lesion at the subcardia or at the gastric fundus, resulting in an approximately vertical angle for the endoscopic knife; **(B)** FRW: Achieving a suitable angle with the lesion, allowing for closer proximity to the lesion.

The FRW technique allowed clear visualization of the lesions in the entire fundus area and the mucosa just below the gastric cardia. (**Figure 3**).

### Operating characteristics of the FRW technique

The FRW technique was mainly used for submucosal injection and lesion dissection during the endoscopic treatment of STMF. However, post-resection trauma suturing was performed in a conventional endoscopy progression.

With the FRW technique, we were able to access and visualize the edge close to the pylorus (7/7), the edge close to the gastric cardia (7/7), and the left and right edges (7/7). The FRW technique can be applied at every site, and no obvious lesion site restriction was observed.

As for the Operational Performance Evaluation of the FRW technique, it was very accessible to navigate the tip of the gastroscope tip left or right using the small adjusting knobs. However, moving the gastroscope forward and backwards still showed moderate difficulty. The gastroscope’s body makes a forward return along the gastric wall, keeping the gastroscopy’s tip stable at all angles due to the support of the gastric wall. The gastroscope was stable at every angle without the need for an assistant to support the endoscope, and the operator could manually adjust the devices in the instrument channel to complete a variety of complex adjustments, cutting, and haemostasis. Since the gastroscopy’s tip could access the lesion’s edges with stable visualization, the details of each layer below the mucosa and above the lesion were clearly observed; however, water accumulation in the lesion area had certain interference. (See **Table 3**).

**Table 3:** Evaluation of FRW manipulating characteristics.

Score Items	Average score	SD
The direction of endoscopy body movement is the same as the direction of observation	2.00	0
The ability to access and observe the lesion	2.00	0
The flexibility of endoscopy body control	1.43	0.54
Stabilization degree of endoscopy body at fixation and movement	2.00	0
Ease of instrument entry and exit:	1.00	0
Hemostatic clamp	0.71	0.49
Hemostatic forceps	0.71	0.49
Submucosal injection needle	2.00	0
Mucosotomy knife	2.00	0

The dissector can be applied parallel to the muscularis propria, forming a good inclination angle during the dissection of the STMF (as shown in the view in **Figure 3B**), except for tumors at the posterior wall of the fundus. There was some resistance while progressing the instruments in the instrument channel using the FRW technique; however, this defect was the same as in the traditional U-turn technique.

### Postoperative and follow-up results

The operation duration using the FRW technique was 47-122 min, and the hospital stay was 7.29 ± 0.49 (7-8) days. Postoperative histopathological evaluation revealed very low-risk stromal tumors in 8 cases and low-risk stromal tumors in 2 cases. No complications were observed, and all patients recovered well after the operation. No residual or recurrent tumors, metastasis, or death occurred during the postoperative follow-ups.

## Discussion

### The FRW technique is a novel direct endoscopy progression method with no paradoxical movement involved

Our team discovered the FRW technique during an STMF procedure. The present study summarises the operation method and success rate of the FRW technique based on practice and analysis of the results. Unlike the traditional progression method, in which the gastroscope’s tip usually reaches the antrum first, the FRW technique uses the resistance of the posterior wall of the upper gastric body (near the junction with the fundus) and the greater curvature as the supporting force to progress the gastroscope along the arc from the posterior wall of the gastric body to the angular incisure, making a forward-return along the gastric wall. This route allows the gastroscope to access the fundus of the stomach and even access just below the gastric cardia (as shown in the view in **Figure 3B**). The fundus of the stomach can then be handled from all angles. As a result, there is no need for paradoxical movement, making it much easier for the operators to access the lesion for more visualization and handling during operation.

Additionally, when observing the fundus of the stomach using the FRW technique, we could clearly visualise the mucosa just below the gastric cardia. The gastroscope’s tip had easy access to the lesion at a close distance, which can significantly benefit operating on lesions below the gastric cardia.

### Solutions to the challenges of endoscopic treatment of GIST in the gastric fundus

The fundus of the stomach is considered a challenging area for ESD (17), ESE, and EFTR (18, 19). Moreover, the ESD technique is complex and prone to perforation (26) because it intentionally destroys the full thickness of the gastric wall, causing perforation to remove the lesion then sutures the wound by nylon suture and or haemostatic clips (27). EFTR is one of the current endoscopic treatments for STMF is EFTR (16), which is why EFTR surgery was predominant in our surgeries (7/7).

Endoscopic operation on the gastric fundus is challenging, mainly due to the need for the traditional endoscopic progression method to be bent backwards (U-turn) to access and observe the fundus of the stomach completely. As a result, the direction of endoscopy body movement is opposite to the direction of observation (paradoxical movement), and the endoscopy body gets suspended, limiting the operating space for the fundus of the stomach (17). Furthermore, when the endoscope’s body is suspended using the traditional U-turn technique, the forward injection force becomes greatly reduced, and the injection needle is perpendicular to the muscularis propria (as shown in the view in **Figure 3A**), making it difficult to find the submucosal space. Following the current commonly used technique, we found that endoscopic treatment of STMF lesions in the middle of the fornix and anterior wall of the fornix was extremely difficult, especially the anterior wall of the fornix at the side of the greater curvature. Thus, a better endoscopic technique was needed to observe, deliver surgical instruments, and operate on these difficult tumor sites.

### The operating characteristics of the FRW technique

The FRW technique solves the problem of avoiding paradoxical movement. Furthermore, the stability score of the endoscopy body was 2 ± 0 points, which was excellent, demonstrating that this technique can achieve non-suspension of the endoscopy body and ensure its stability. Additionally, the handling of the endoscopy body and devices in the channel would not be significantly affected by respiratory and heartbeat beats. Finally, the FRW technique allowed easy access to the lesion and its observation.

The ease of device entry and exit using the FRW technique in the 7 patients was 1 ± 0 points, suggesting that the FRW technique had a certain impact on device entry and exit. The injection needle and mucosotomy knife were not affected (2 ± 0 points), while hemostatic forceps and hemostatic clips were significantly affected (0.71 ± 0.49 points). In 2 patients, the hemostatic forceps and hemostatic clips could not pass through the curved channel, and we had to complete the pre-insertion of the device by unbending the endoscopy body and then bending it again to reach the lesion. The difficulty of the passage of the device is mainly related to the long rigid part at the front end of the device and the large bending at the channel.

The flexibility score of the endoscopy body when using the FRW technique was 1.43 ± 0.54 points, but the movement coordination and comfort of the endoscopy body were still somewhat hindered. At present, the synchronization of front and back movement of the endoscopy body is relatively unaffected, but the accuracy is slightly affected. Furthermore, the small knob swing affects the left-right movement and angle adjustment of the endoscopy body.

### Factors affecting the FRW technique

The success rate of the FRW technique was 70% in our clinical practice. Currently, the FRW technique can only be performed while patients are in the supine position and under general anaesthesia with endotracheal intubation. In the three cases where the FRW technique failed, we tried gastric cavity morphology after various gas volume adjustments and postural changes, including left lateral decubitus and supine positions, but FRW was still not applicable. Whether the failure cases were due to specific characteristics of the gastric fundus or other factors remains to be explored through a larger sample size. The present study had some limitations due to the small sample of STMF patients who qualified for the FRW technique. Therefore, more large-scale observational studies are needed to further validate the FRW technique. Additionally, this study was not a randomised clinical trial with a large sample size since GIST is an uncommon disease. In future, we plan to conduct clinical trials for the FRW technique to confirm its reliability.

## Conclusions

In summary, the clinical application of the FRW technique can greatly benefit the endoscopic treatment of gastric fundus stromal tumors. This method has distinct advantages in terms of accessing and observing the lesion, increasing the endoscopy body stability, and matching the endoscopy body movement with the direction of observation.

## Data availability statement

The original contributions presented in the study are included in the article/**Supplementary Material**. Further inquiries can be directed to the corresponding author.

## Ethics statement

The studies involving human participants were reviewed and approved by Ethics Committee of The Fourth Affiliated Hospital of China Medical University. Written informed consent for participation was not required for this study in accordance with the national legislation and the institutional requirements.

## Author contributions

HM-G and YS collected the data and wrote the manuscript. SC, FM, YZ, and YY collected the data and revised the manuscript. Z-FZ was the leading surgeon who completed the procedures in this manuscript. He also revised the manuscript. LL checked all data and revised the manuscript. All authors contributed to the article and approved the submitted version.
